# Biomass and mineral nutrient partitioning among self-pollinated and cross-pollinated fruit on the same strawberry plant

**DOI:** 10.1371/journal.pone.0269485

**Published:** 2022-06-03

**Authors:** Cao Dinh Dung, Helen M. Wallace, Shahla Hosseini Bai, Steven M. Ogbourne, Stephen J. Trueman

**Affiliations:** 1 Centre for Bioinnovation, University of the Sunshine Coast, Sippy Downs, Queensland, Australia; 2 School of Science, Technology and Engineering, University of the Sunshine Coast, Sippy Downs, Queensland, Australia; 3 Potato, Vegetable and Flower Research Center–Institute of Agricultural Science for Southern Viet Nam, Da Lat, Lam Dong, Viet Nam; 4 Centre for Planetary Health and Food Security, School of Environment and Science, Griffith University, Brisbane, QLD, Australia; University of Delhi, INDIA

## Abstract

Pollen-parent effects on fruit size and quality have been found previously among competing self-pollinated and cross-pollinated fruit on the same Redlands Joy strawberry plant. These effects occur independently of the percentage of fertilized seeds on the fruit, but the expression of these effects on fruit size and some aspects of quality are greatest when calcium is in shortest supply. Here, we aimed to clarify at what developmental stages the self-pollinated and cross-pollinated fruit diverge in size and quality and whether differences between self-pollinated and cross-pollinated fruit are due to early differences in nutrient accumulation. Fruit were harvested at 1, 2 and 3 weeks after hand-pollination and at full ripeness, approximately 4 weeks after hand-pollination. We measured fruit mass, length, diameter, colour, and the concentrations of aluminium, boron, calcium, copper, iron, nitrogen, magnesium, manganese, sodium, phospho-rous, potassium and zinc. Temporary increases in fruit mass, length or diameter due to cross-pollination were evident at 1 or 2 weeks after pollination. Consistent increases in size and skin darkness from cross-pollination emerged in the final week of fruit development. We found little evidence that self-pollinated and cross-pollinated fruit differed in mineral nutrient accumulation at any stage of fruit development. The results demonstrate that cross-pollination effects on strawberry fruit size are evident briefly during early fruit growth but emerge mainly during the final week of fruit development. The effects of cross-pollination on fruit size are not the result of early differences in mineral nutrient accumulation between self-pollinated and cross-pollinated fruit.

## Introduction

Xenia refers to the effects of different pollen parents on characteristics of the seed and fruit including size, shape, colour, developmental timing and chemical composition [1]. Plant cultivars differ greatly in their degree of self-compatibility, and these differences may affect the extent to which cross-pollination affects fruit quality [2]. Cross-pollination, in addition, can increase fruit mass, yield and quality of many horticultural crops including almond, blueberry, grape, guava, kiwifruit, macadamia and mandarin, when compared with self-pollination [3–10]. Furthermore, cross-pollinated fruit or seeds out-compete self-pollinated fruit or seeds that are growing on the same almond, feijoa, *Banksia spinulosa* or *Phormium tenax* plant, resulting in the cross-pollinated fruit or seeds having greater mass or size [11–14].

Most strawberry cultivars are considered self-compatible although some cultivars possess some degree of self-incompatibility [15]. Cross-pollinated and self-pollinated mature fruit of the self-compatible strawberry cultivar, Redlands Joy, do not differ significantly in size when they develop on different plants [15]. However, xenia effects on mature fruit size become apparent when cross-pollinated and self-pollinated Redlands Joy fruit compete on the same plant, and these xenia effects are amplified when calcium is in shortest supply [16]. Xenia effects on strawberry fruit growth are considered to result from differences in auxin production by developing seeds [1, 17]. Lower auxin concentrations are often found in self-pollinated fruit than cross-pollinated fruit [1, 17–19]. The differences in auxin concentration between self-pollinated fruit and cross-pollinated fruit have been thought to directly affect mineral nutrient influx to the fruit [1, 20, 21], with cross-pollinated apple and strawberry fruit having higher concentrations of some mineral nutrients than self-pollinated fruit [1, 21, 22]. Therefore, cross-pollination might provide health benefits for consumers. The effects of pollen source on auxin production by strawberry fruit occur soon after the seeds are formed [18] and mineral nutrient accumulation commences during the early, green stages of fruit development [23]. The developmental stage at which the pollen source begins to affect fruit mass, and whether the effects of pollen source on fruit mass are the result of differences in mineral nutrient accumulation, remain unclear for berry fruit.

In this study, Redlands Joy strawberry was used as a model crop to evaluate the effects of pollen source on fruit mass, fruit size and the accumulation of mineral nutrients. Self-pollinated and cross-pollinated fruit on the same plant were harvested at 1, 2 and 3 weeks after hand-pollination and at full ripeness. It was hypothesized that cross-pollinated fruit would grow faster than self-pollinated fruit during the early stages of fruit development and that this difference in growth would be related to differences in mineral nutrient accumulation. We aimed to clarify: 1) at what developmental stages xenia effects on fruit mass, size and colour emerge; and 2) whether differences in fruit mass and size are related to early differences in mineral nutrient accumulation between cross-pollinated and self-pollinated fruit.

## Materials and methods

### Plant material

Two strawberry cultivars, Redlands Joy and Rubygem, were used for this study. These cultivars were selected for commercial cultivation under subtropical conditions in eastern Australia [24–28]. Rooted runners of each cultivar were obtained from Sweets Strawberry Runners (Stanthorpe, Australia) in May 2018. Rooted runners were approximately 20 cm in height and 1.5 cm in crown diameter. We transplanted each rooted runner into a 4.5 L pot filled with coco-peat (EC < 1mS/cm, pH = 5.5–7.0) and perlite (4:1, v:v) plus 2.5 g of Osmocote fertilizer (N:P:K = 19.6:16.0:5.0% w/w, plus trace elements) (Scotts International, Heerlen, The Netherlands). We placed the pots in a glasshouse at the University of the Sunshine Coast, Sippy Downs, Australia (26°43’S 153°03’E). Plant spacing was 20 cm from pot to pot [28, 29]. Each plant was top-dressed monthly with 15 g of Osmocote fertilizer and sprayed weekly with 5 mL of 1% (v/v) aqueous PowerFeed® foliar fertilizer (Seasol International, Bayswater, Australia) during establishment from transplanting until the commencement of hand-pollinations. Water was applied manually to the plant canopy using a water hose to avoid wetting flowers during the pollination period. Pots were rotated regularly to minimize differences in long-term light interception among different sides of each plant. Runners and senesced leaves were removed frequently.

### Experimental design

Redlands Joy plants were arranged into three experiments, with 10 plants per experiment. The three experiments (a–c) were undertaken under different levels of calcium crop nutrition at (a) 1, (b) 2 or (c) 4 kg elemental Ca ha^-1^ spray^-1^, respectively. Each plant received eight sprays of calcium as Grotek Cal-Max (GS Distribution, Langley, Canada), with approximately 5 mL of solution at each spray. This volume ensured that each leaf on each plant was totally covered with Ca solution. Each plant received (a) 2, (b) 4 or (c) 8 mg Ca mL^-1^ at each spray, depending on the experiment. We performed the first spray at first flower petal fall. The second spray was performed 1 week later, and subsequent sprays were applied at 2-week intervals.

Every flower on each experimental plant was allocated to one of two pollination treatments: (1) pollination by Redlands Joy (Self) or (2) pollination by Rubygem (Cross). Successive flowers were either self-pollinated or cross-pollinated, creating a 50/50 mixture of self-pollinated and cross-pollinated fruit on each plant. The pollination treatment applied to the first flower on each plant (i.e. Self or Cross) was rotated successively from one experimental plant to the next, so that the first flower of five plants in each experiment was self-pollinated while the first flower of the other five plants was cross-pollinated. Details of the pollen collection and hand-pollination methods have been provided previously [15, 16]. Briefly, each flower on each experimental plant had its anthers removed before the flower opened and was then covered with a fine-mesh bag until the stigma was receptive. Mature anthers of each cultivar were harvested daily and dried at glasshouse temperature from 1200 H to 1400 H in the lid of a 100-mL plastic jar. Released pollen was then transferred to the plastic jars, which were closed and stored overnight in a refrigerator at 4°C. Hand-pollination was performed from 0700 H to 1200 H. Flowers on each experimental plant were hand-pollinated using a paintbrush, with separate brushes used for each pollen-donor cultivar. Each flower was labelled with a miniature paper tag that recorded the pollination treatment and the date of first hand-pollination and was covered again with a fine-mesh bag to completely exclude other pollen. Pollination was repeated daily until the petals started to fall.

### Fruit quality

**Fig 1:**
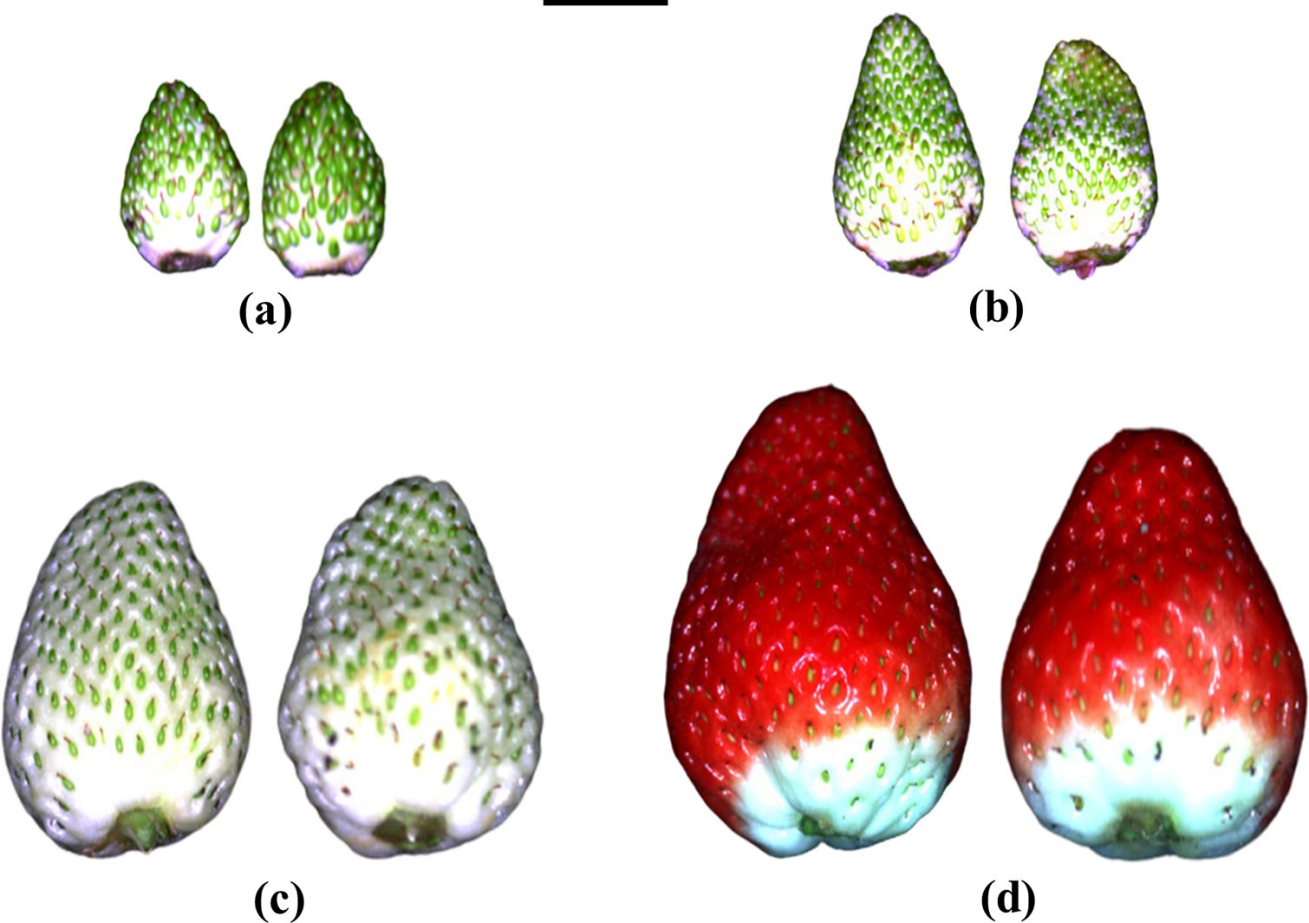
Cross-pollinated fruit (left) and self-pollinated fruit (right) of Redlands Joy strawberry under 2 kg Ca ha^-1^ spray^-1^ that were harvested at (a) 1, (b) 2, and (c) 3 weeks after first hand-pollination and (d) at full ripeness. Scale bar = 1 cm. Photographs: Cao Dinh Dung.

**Fig 2:**
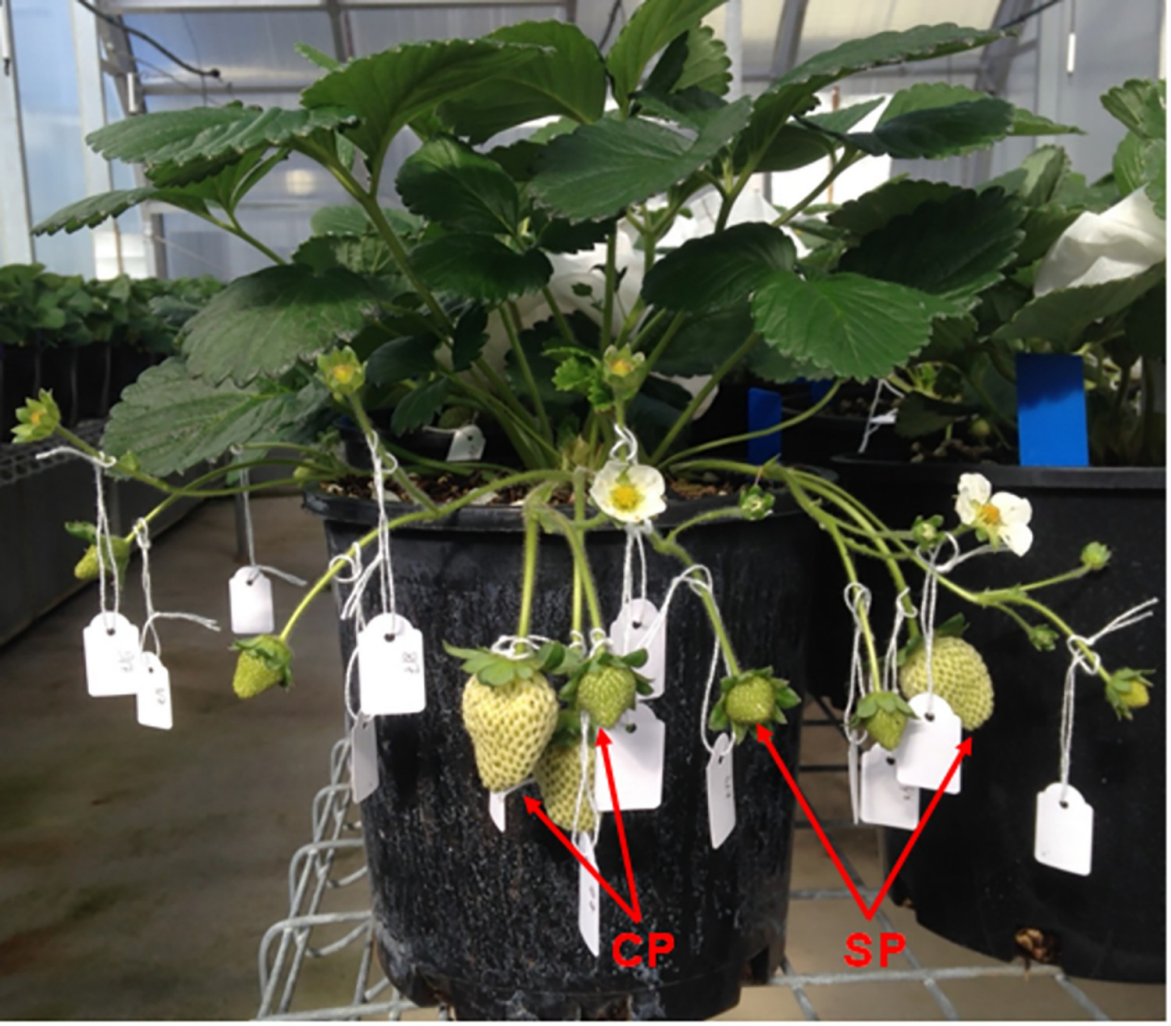
Cross-pollinated fruit (CP, left) and self-pollinated fruit (SP, right) on the same Redlands Joy strawberry plant at 1 week (smaller fruit) and 2 weeks (larger fruit) after first hand-pollination. Photograph: Cao Dinh Dung.

Fruit were harvested at 1, 2 or 3 weeks after first hand-pollination or at commercial maturity when the fruit became fully red (Fig 1). Commercial maturity occurred approximately 4 weeks after first hand-pollination. The fruit of each developmental stage were numbered consecutively within each pollination treatment according to the date of harvest. Fruit harvested at 1, 2 or 3 weeks after pollination on each plant (Fig 2) were harvested on the same day near the end of each fruiting cycle, using the date of hand-pollination to determine the age of each fruit. Ripe fruit were harvested when they became fully red. The total numbers of self-pollinated plus cross-pollinated fruit harvested per plant were 6–10, 10–26, 12–32 and 22–50 at 1, 2 and 3 weeks after pollination and at full ripeness, respectively, with an equal number of fruit for each pollination treatment. We recorded the fresh mass, length and diameter of each fruit, excluding the pedicel and sepals. Colour was assessed for each fruit using a CR-10 colorimeter (Konica Minolta, Chiyoda, Japan) in the L*a*b* colour space. L*, a* and b* values indicate brightness, redness and yellowness, respectively. Fruit were then stored at -20°C.

Three frozen cross-pollinated and three frozen self-pollinated fruit within each developmental stage were then selected to create a composite sample from each plant for mineral nutrient analysis. Fruit 1, 2 and 3 (within each developmental stage) from each pollination treatment were used at 1 week after pollination, while fruit 1, 3 and 5 (within each developmental stage) from each pollination treatment were used at 2 and 3 weeks after pollination and at full ripeness. Nitrogen concentrations were determined by combustion analysis using a LECO 928 analyser (LECO, Saint Joseph, MI) [30–32]. Aluminium, boron, calcium, copper, iron, magnesium, manganese, phosphorus, potassium, sodium and zinc concentrations were determined by inductively coupled plasma atomic emission spectroscopy [33].

### Leaf mineral nutrient concentrations across the three experiments

We sampled one leaf per plant each month when the leaf opened fully, with a total of five leaves being sampled per plant over 4 months. The first sample immediately preceded the first foliar calcium application. We dried each leaf at 70°C for 24 h. Mineral nutrient concentrations of each leaf were analysed using the same methods used for fruit samples, and the nutrient concentrations of each of the five leaves were used to calculate an average concentration of each nutrient for each plant.

### Statistical analysis

Data were analysed using IBM SPSS Statistics v. 26. Mineral nutrient concentration and content data for fruitlets and fruit were analysed by paired t-tests. Mineral nutrient concentration data for leaves were analysed by 1-way analyses of variance (ANOVA) followed by Tukey’s Honestly Significant Difference (HSD) test when significant differences were detected by ANOVA. Fruit quality data were analysed using generalized linear models (GLMs). Pollination, plant, and fruit number nested within plant were regarded as main factors. Treatment differences were regarded as significant at P < 0.05. Means are presented with standard errors.

## Results

### Biomass and leaf mineral nutrient concentrations across experiments

The foliar concentrations of most mineral nutrients did not differ significantly across experiments (Table 1). However, Ca concentration was higher for plants in the experiment receiving 4 kg Ca ha^-1^ spray^-1^ than for plants in the experiment receiving 1 kg Ca ha^-1^ spray^-1^, and P concentration was higher and Al and Cu concentrations were lower for plants receiving 4 kg Ca ha^-1^ spray^-1^ than plants receiving either 1 or 2 kg Ca ha^-1^ spray^-1^ (Table 1).

**Table 1:** Foliar mineral-nutrient concentrations (mg kg^-1^ dry mass) of Redlands Joy strawberry plants under three levels of calcium nutrition.

Nutrient	Calcium level		
	1 kg Ca ha^-1^ spray^-1^	2 kg Ca ha^-1^ spray^-1^	4 kg Ca ha^-1^ spray^-1^
**Calcium (Ca)**	**5851 ± 221 b**	**6114 ± 240 ab**	**6883 ± 220 a**
**Nitrogen (N)**	31928 ± 627 a	31739 ± 647 a	32734 ± 860 a
**Phosphorus (P)**	**3262 ± 162 b**	**3185 ± 124 b**	**3820 ± 161 a**
**Potassium (K)**	19037 ± 755	19463 ± 457	19340 ± 436
**Aluminium (Al)**	**21.7 ± 0.8 a**	**20.2 ± 1.1 a**	**16.0 ± 0.5 b**
**Boron (B)**	54.1 ± 1.7 a	58.6 ± 3.5 a	60.7 ± 1.4 a
**Copper (Cu)**	**3.45 ± 0.30 a**	**3.36 ± 0.28 a**	**1.77 ± 0.11 b**
**Iron (Fe)**	72.8 ± 3.1 a	84.4 ± 7.6 a	72.0 ± 3.0 a
**Magnesium (Mg)**	3702 ± 11 a	3756 ± 11 a	3832 ± 11 a
**Manganese (Mn)**	202 ± 14 a	217 ± 11 a	221 ± 15 a
**Sodium (Na)**	36.2 ± 2.1 a	30.6 ± 2.1 a	30.5 ± 2.9 a
**Zinc (Zn)**	23.5 ± 0.6 a	24.1 ± 0.4 a	25.4 ± 0.8 a

Means ± SE with bold font and different letters within a row are significantly different (ANOVA and Tukey’s HSD test; P < 0.05; n = 10 plants).

### Cross-pollination effects on fruit growth and colour

**Fig 3:**
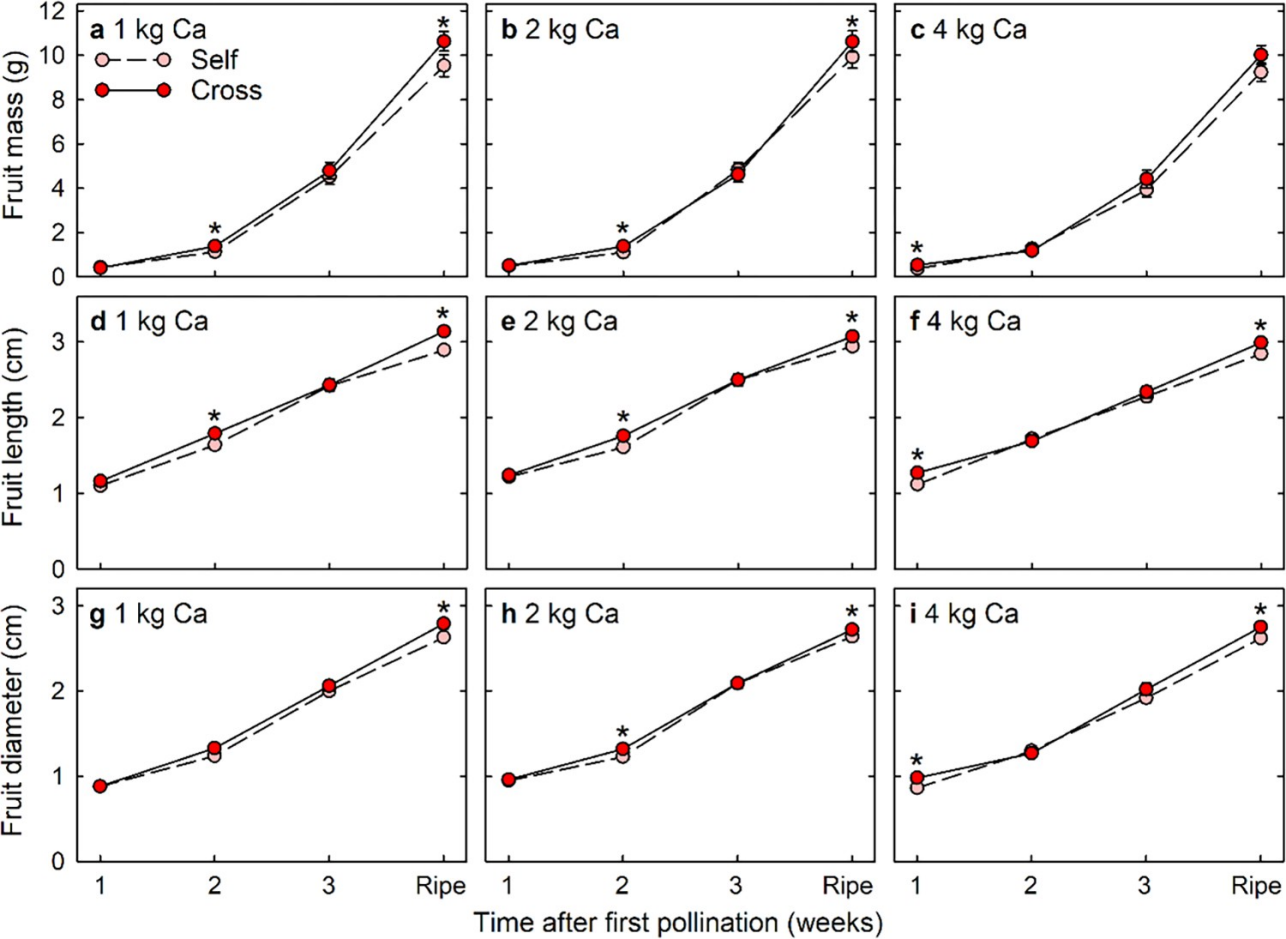
(a–c) Fresh mass, (d–f) length and (g–i) diameter of Redlands Joy strawberry fruit arising from self-pollination (Self) or cross-pollination by cultivar Rubygem (Cross) under three levels of calcium nutrition: (a, d, g) 1 kg, (b, e, h) 2 kg or (c, f, i) 4 kg Ca ha^-1^ spray^-1^. Means ± SE with an asterisk within a calcium level and time point are significantly different (GLM; P < 0.05; n = 65–82, 105–117, 123–129 and 287–336 fruit for fruit harvested at 1, 2 and 3 weeks after pollination and at full ripeness respectively under three levels of calcium nutrition).

Cross-pollinated fruit were heavier (Fig 3A–3C), longer (Fig 3D–3F) and sometimes broader (Fig 3G–3I) than self-pollinated fruit at either 1 or 2 weeks after first pollination, with the timing of this difference being dependent on the calcium level. Cross-pollinated and self-pollinated fruit did not differ significantly in mass, length or diameter at 3 weeks after first pollination. Cross-pollinated fruit had consistently greater length and diameter than self-pollinated fruit at ripeness, approximately 4 weeks after first pollination. More than half of the total biomass accumulation occurred in the final week of fruit development (Fig 3).

**Fig 4:**
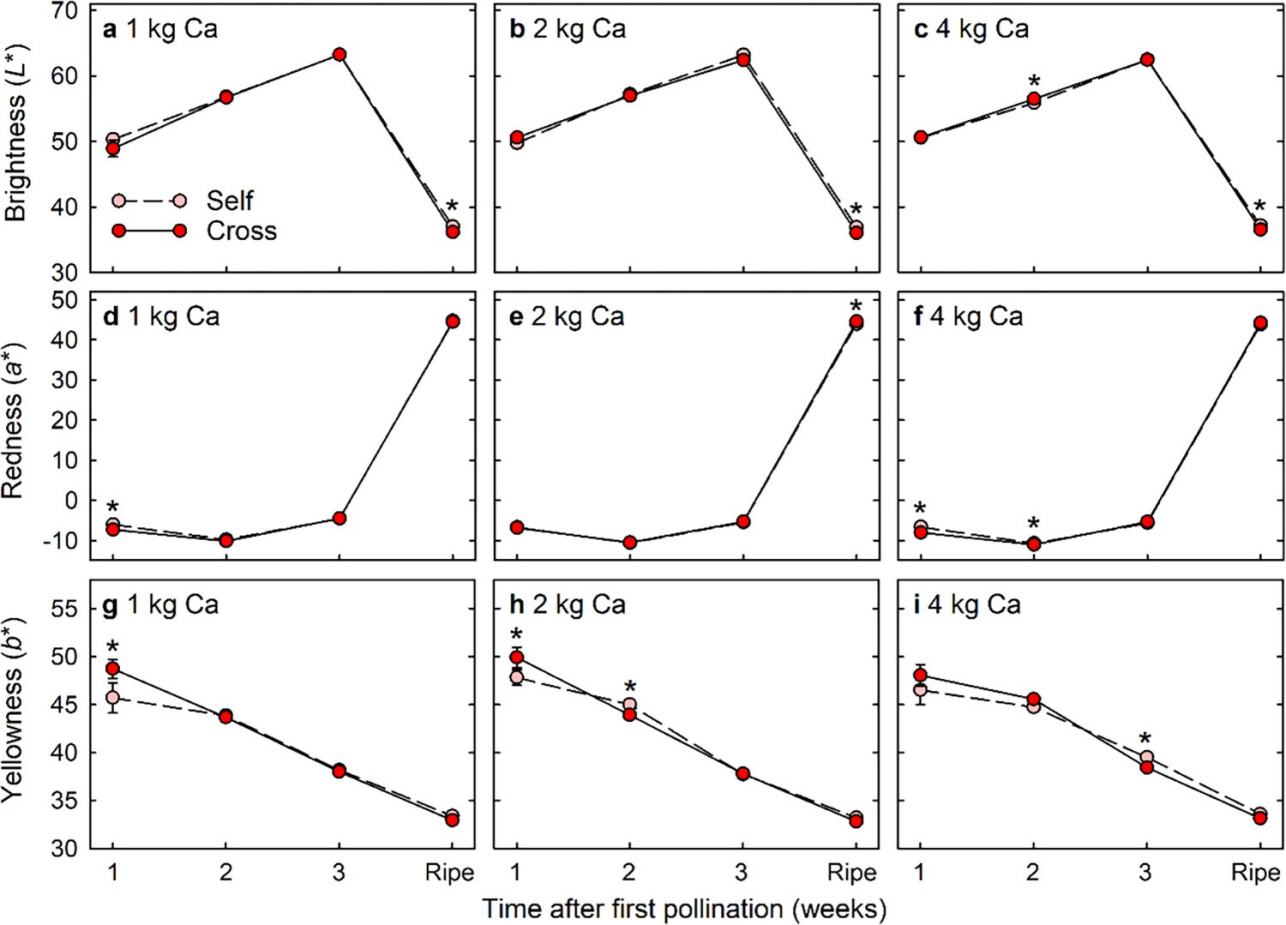
(a–c) Brightness (L*), (d–f) redness (a*), and (g–i) yellowness (b*) of Redlands Joy strawberry fruit arising from self-pollination (Self) or cross-pollination by cultivar Rubygem (Cross) under three levels of calcium nutrition: (a, d, g) 1 kg, (b, e, h) 2 kg or (c, f, i) 4 kg Ca ha^-1^ spray^-1^. Means ± SE with an asterisk within a calcium level and time point are significantly different (GLM; P < 0.05; n = 55–76, 105–117, 123–129 and 287–334 fruit for fruit harvested at 1, 2 and 3 weeks after pollination and at full ripeness respectively under three levels of calcium nutrition).

Cross-pollinated and self-pollinated fruit differed little in brightness (Fig 4A–4C), redness (Fig 4D–4F) or yellowness (Fig 4G–4I) during the first 3 weeks after first pollination, and effects of pollen source on fruit appearance were not consistent across calcium levels or sampling times during this period. Fruit darkened and developed their red colour during the final week of development, at which time the cross-pollinated fruit became consistently darker than self-pollinated fruit. Redness and yellowness of ripe fruit generally did not differ significantly between pollination treatments, except that cross-pollinated fruit were redder than self-pollinated fruit on plants that received 2 kg Ca ha^-1^ spray^-1^.

### Cross-pollination effects on fruit mineral-nutrient accumulation

**Fig 5:**
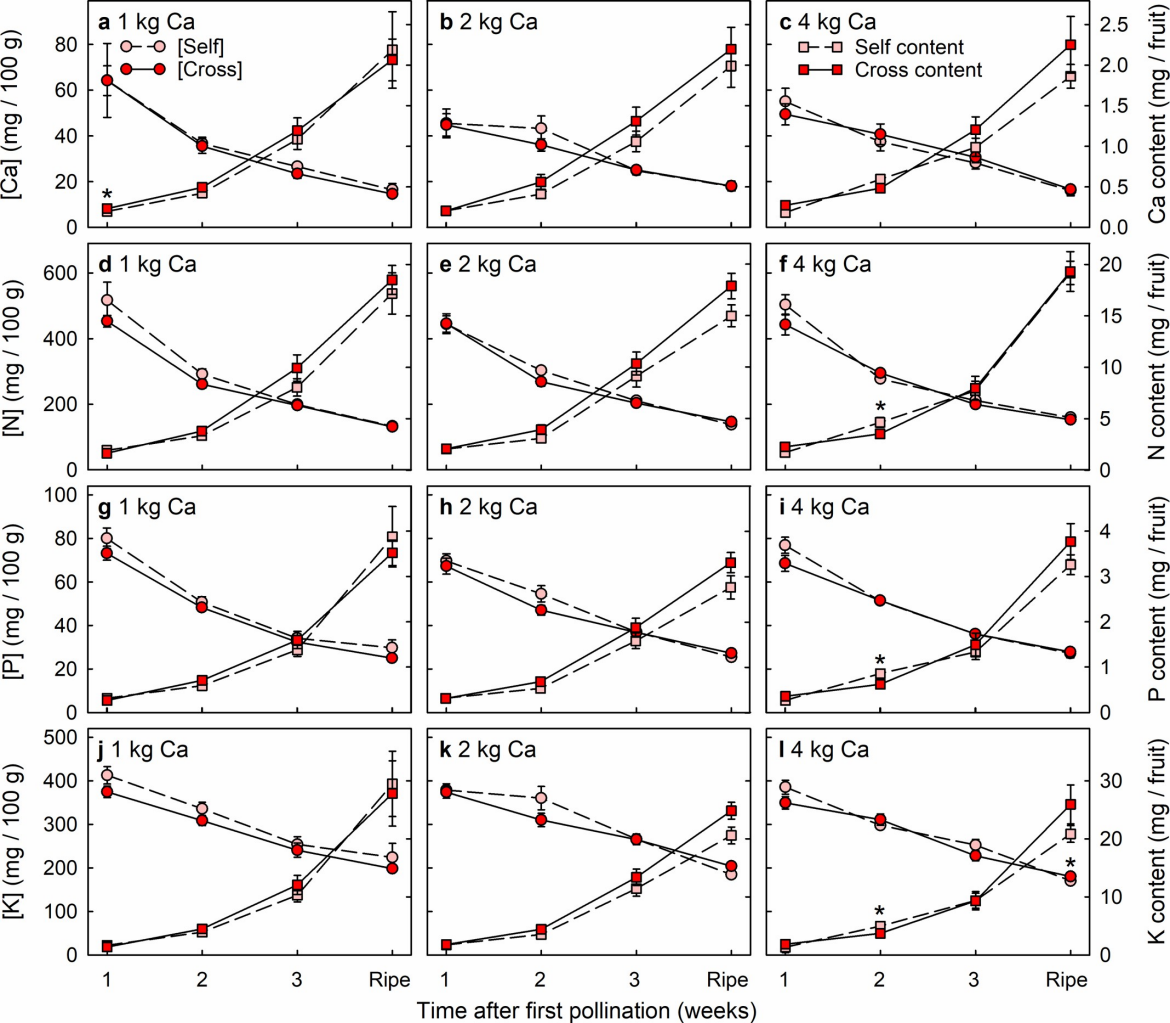
Concentration (circles) and content (squares) of (a–c) calcium (Ca), (d–f) nitrogen (N), (g–i) phosphorus (P) and (j–l) potassium (K) of Redlands Joy strawberry fruit arising from self-pollination (Self) or cross-pollination by cultivar Rubygem (Cross) under three levels of calcium nutrition: (a, d, g, j) 1 kg, (b, e, h, k) 2 kg or (c, f, i, l) 4 kg Ca ha^-1^ spray^-1^. [Self] and [Cross] refer to nutrient concentrations in Self and Cross fruit, respectively. An asterisk (*) indicates that the two subtending means ± SE are significantly different (paired t-test; P < 0.05; n = 10 plants).

**Fig 6:**
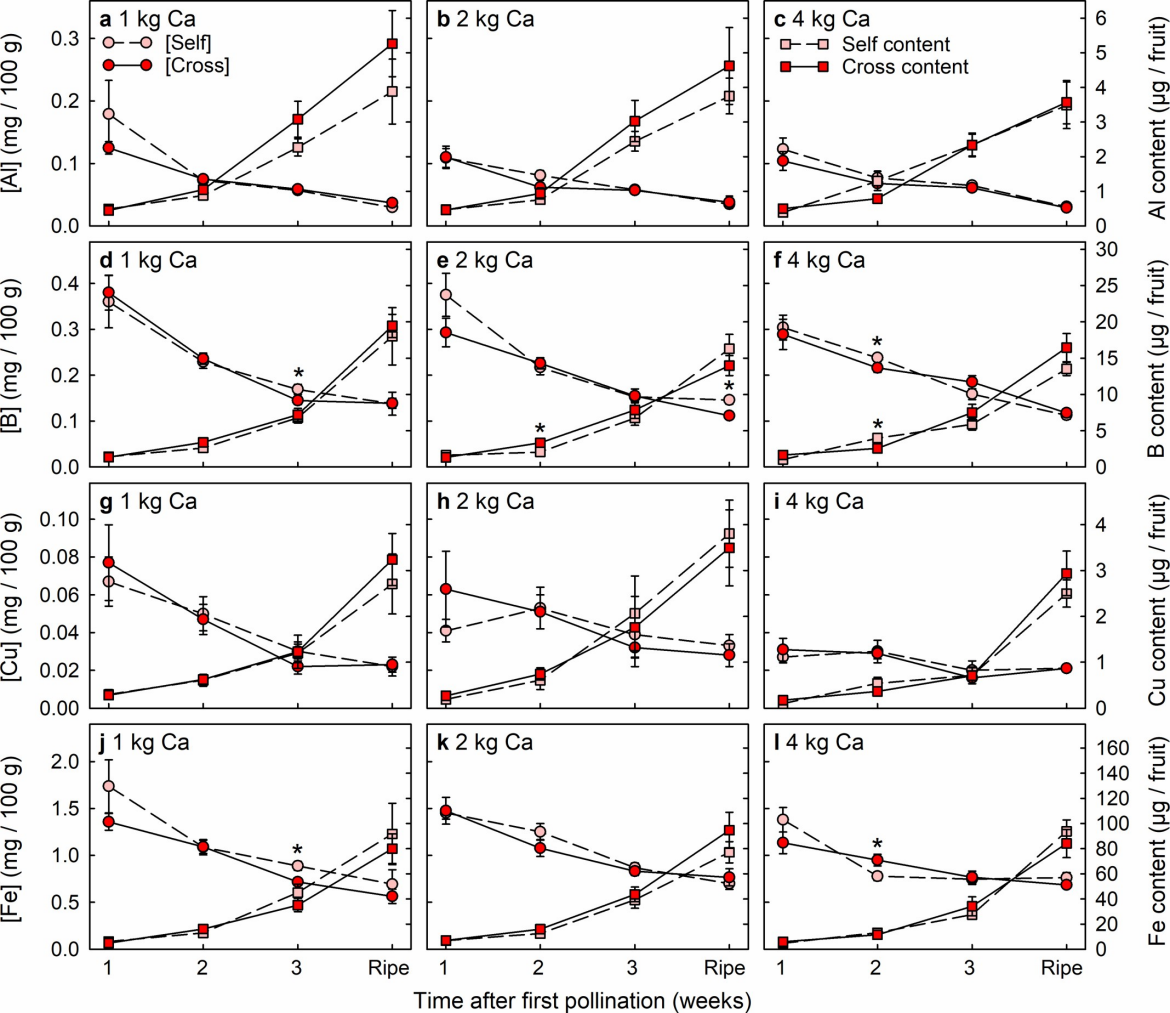
Concentration (circles) and content (squares) of (a–c) aluminium (Al), (d–f) boron (B), (g–i) copper (Cu) and (j–l) iron (Fe) of Redlands Joy strawberry fruit arising from self-pollination (Self) or cross-pollination by cultivar Rubygem (Cross) under three levels of calcium nutrition: (a, d, g, j) 1 kg, (b, e, h, k) 2 kg or (c, f, i, l) 4 kg Ca ha^-1^ spray^-1^. [Self] and [Cross] refer to nutrient concentrations in Self and Cross fruit, respectively. An asterisk (*) indicates that the two subtending means ± SE are significantly different (paired t-test; P < 0.05; n = 10 plants).

**Fig 7:**
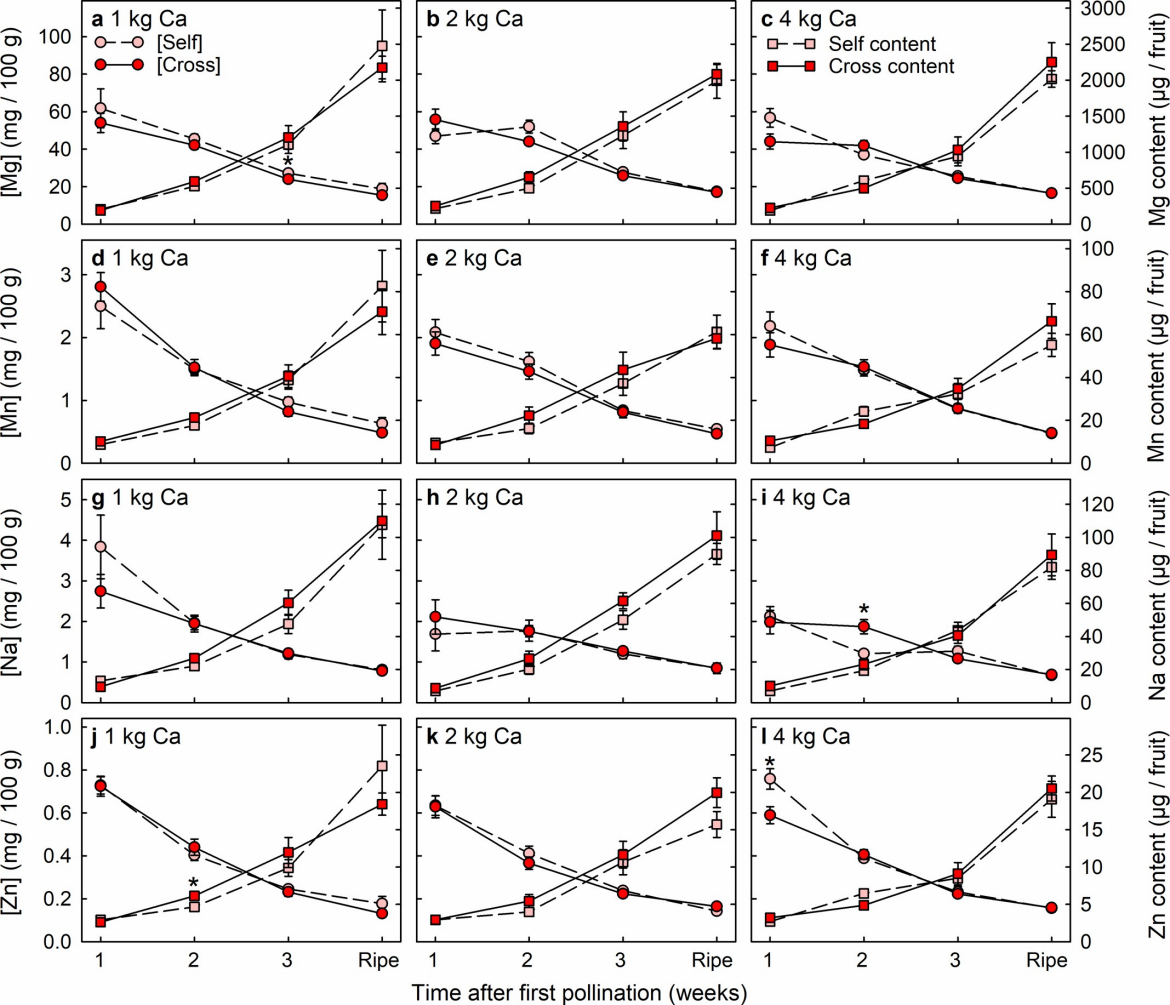
Concentration (circles) and content (squares) of (a–c) magnesium (Mg), (d–f) manganese (Mn), (g–i) sodium (Na) and (j–l) zinc (Zn) of Redlands Joy strawberry fruit arising from self-pollination (Self) or cross-pollination by cultivar Rubygem (Cross) under three levels of calcium nutrition: (a, d, g, j) 1 kg, (b, e, h, k) 2 kg or (c, f, i, l) 4 kg Ca ha^-1^ spray^-1^. [Self] and [Cross] refer to nutrient concentrations in Self and Cross fruit, respectively. An asterisk (*) indicates that the two subtending means ± SE are significantly different (paired t-test; P < 0.05; n = 10 plants).

Cross-pollinated and self-pollinated fruit rarely differed significantly in the concentrations or contents of mineral nutrients (Figs 5–7). Only 16 significant differences in mineral nutrient levels were detected among 288 comparisons between pollination treatments, i.e. < 6% of cases. These 16 differences were dispersed across nine of the 12 nutrients (Ca, N, P, K, B, Fe, Mg, Na and Zn), without nutrient levels being consistently higher in either the cross-pollinated fruit or the self-pollinated fruit. Nutrient concentrations declined greatly across the period of fruit development (Figs 5–7), except for Cu concentration in fruit from plants that received 4 kg Ca ha^-1^ spray^-1^ (Fig 6I). None the less, fruit accumulated significant amounts of all nutrients during the final week of development.

## Discussion

A xenia effect on fruit growth was found briefly within 1 or 2 weeks of pollination among cross-pollinated and self-pollinated fruit on Redlands Joy strawberry plants. These results confirm our first hypothesis that xenia effects on fruit growth occur at early stages of fruit development, although consistent differences in fruit size only emerged during the final week of fruit development. Cross-pollinated fruit rarely differed significantly from self-pollinated fruit in the concentrations or contents of mineral nutrients. These results lead us to reject our second hypothesis that differences in fruit mass are related to differences in mineral nutrient accumulation.

Cross-pollinated fruit were heavier and longer in shape than self-pollinated fruit at either 1 or 2 weeks after pollination, but no significant differences in fruit mass, length or diameter were evident at 3 weeks after pollination. More than half of the fruit mass accrued during the final week of fruit development, at which time the cross-pollinated fruit became both longer and broader than self-pollinated fruit. Pollen-parent effects on fruit size have been found in many fruit crops such as blueberry [34], strawberry [17, 18] and pear [19], and xenia effects on fruit size are thought to be caused by differences in auxin production by the fertilized seeds [1, 17–19]. Cross-pollinated seeds often produce more auxin than self-pollinated seeds [18]. Cross-pollinated strawberry fruit possess higher indole-3-acetic acid (IAA) concentrations than self-pollinated fruit during the early stages of development and, thus, cross-pollinated fruit sometimes develop faster than self-pollinated fruit [17, 18]. Xenia effects on blueberry, strawberry and pear fruit size [17–19, 34] were evaluated when self-pollinated fruit and cross-pollinated fruit developed on different plants. We have recently demonstrated that xenia effects on the size of mature fruit of self-compatible Redlands Joy do not occur when self-pollinated and cross-pollinated develop on different plants [15]. However, xenia effects on mature fruit size become apparent when self-pollinated and cross-pollinated fruit compete on the same Redlands Joy plant [16]. In the current study, we have clarified that some xenia effects on fruit size occur at early stages of fruit development, but consistent differences in fruit size only emerge in the final week of fruit development. Pollen-parent effects on macadamia nut mass are also sometimes evident during the early stages of fruit development, but most effects emerge about half-way through fruit development when the shell hardens and the nut reaches its final diameter [35]. Pollen-parent effects on hickory fruit mass and size also emerge half-way through fruit development, with cross-pollinated fruit becoming heavier and larger than self-pollinated fruit [36]. Consistent xenia effects on strawberry fruit colour emerged only during the final week of fruit development. Redlands Joy fruit developed their dark red colour during this final week, with cross-pollinated fruit becoming darker at this stage than self-pollinated fruit. Pollen parentage often affects fruit colour [1], e.g. cross-pollination increases darkness and redness of kiwifruit and pear fruit [6, 37].

Foliar mineral nutrient concentrations were generally similar between the three experiments where plants were treated with either 1, 2 or 4 kg calcium ha^-1^ spray^-1^. However, plants that received 4 kg calcium ha^-1^ spray^-1^ had higher foliar calcium and phosphorus concentrations and lower aluminium and copper concentrations. Sufficient calcium, phosphorus and copper concentrations in leaves of Tudla strawberry range from 7,700–14,800 mg kg^-1^, 2,000–3,800 mg kg^-1^ and 3.0–22.5 mg kg^-1^, respectively [38]. Therefore, foliar calcium levels could be considered slightly deficient, while phosphorus levels would be sufficient, if similar concentrations were required by Redlands Joy plants. Foliar calcium applications do not increase the foliar concentrations of mineral nutrients in Smoothee Golden Delicious apple [39]. However, calcium sprays increase leaf dry matter and chlorophyll content in Oso Grande and Camarosa strawberry [40]. Leaf dry matter and chlorophyll content can be positively correlated with leaf phosphorus concentrations [41]. The highest rate of calcium application (i.e. 4 kg ha^-1^ spray^-1^) led to copper deficiency and produced fruitlets with low copper concentrations during early fruit development. Calcium supply can reduce the uptake of aluminium and copper [42–44]. Aluminium has very minor effects on strawberry plant growth and fruit development, but copper deficiency can decrease nitrogen fixation and reduce strawberry fruit size and firmness [42, 43, 45, 46].

Cross-pollinated and self-pollinated strawberry fruit rarely differed in mineral nutrient concentrations or contents, either during early fruit development or at maturity. Pollen parents can affect fruit chemical composition [1, 10, 47] and cross-pollination increases calcium concentrations in apple and avocado fruit [21, 22, 47]. The accumulation of mineral nutrients to fruit has been thought to be affected directly by the auxin levels produced by developing seeds [1, 20, 21]. Auxin production and mineral nutrient accumulation by strawberry fruit commence during the early stages of fruit development [18, 23]. Thus, we had hypothesized that xenia effects on fruit size were related to early differences in nutrient accumulation between cross-pollinated and self-pollinated fruitlets. This may have been the case if increased hormone production by cross-pollinated seeds led directly to increased nutrient uptake. Instead, growth differentials between cross-pollinated and self-pollinated fruit occurred independently of nutrient accumulation, such that nutrient flow into the fruit followed water transport rather than regulated water transport. Our findings support the current scientific understanding of nutrient transport, which is that mineral nutrient translocation to fruit via the xylem and phloem occurs by mass flow of water [48–51]. Fruit size is possibly regulated by the amount of water uptake, which may be affected by sugar concentrations as sugar contributes significantly to osmotic strength and water uptake. We have shown in our two previous studies that cross-pollinated ripe fruit often have higher Brix:acid ratio, but not higher Brix, than self-pollinated fruit [15, 16]. However, sugar levels in the young developing fruit require further investigation. Fruit size was regulated by the paternity of developing seeds, and xenia effects on fruit size can occur immediately after fertilization [1]. Xenia effects on strawberry fruit size are related to water accumulation by the fruit, and it is the mass flow of water that regulates mineral nutrient influx [50, 51]. Strawberry growers should consider interplanting different cultivars, managing insect pollinators such as bees on farms, and supplying water efficiently during early fruit development to ensure the best possible fruit quality.

## Conclusion

A xenia effect on strawberry fruit growth was evident briefly at early stages of fruit development. However, most fruit biomass accumulation and colour development occurred in the final week of fruit development, at which time cross-pollinated fruit became larger and darker than self-pollinated fruit. The differences in size between cross-pollinated and self-pollinated fruit were not the result of significant differences in mineral nutrient accumulation. Instead, nutrient transport to early-developing strawberry fruit followed rather than regulated water flow, consistent with current theories of nutrient transport through xylem and phloem.
